# A Multivariable Probability Density-Based Auto-Reconstruction Bi-LSTM Soft Sensor for Predicting Effluent BOD in Wastewater Treatment Plants

**DOI:** 10.3390/s24237508

**Published:** 2024-11-25

**Authors:** Wenting Li, Yonggang Li, Dong Li, Jiayi Zhou

**Affiliations:** School of Automation, Central South University, Changsha 410083, China; liwenting@csu.edu.cn (W.L.);

**Keywords:** effluent BOD, soft sensor, Bi-LSTM, MPDAR strategy, wastewater treatment

## Abstract

The precise detection of effluent biological oxygen demand (BOD) is crucial for the stable operation of wastewater treatment plants (WWTPs). However, existing detection methods struggle to meet the evolving drainage standards and management requirements. To address this issue, this paper proposed a multivariable probability density-based auto-reconstruction bidirectional long short-term memory (MPDAR-Bi-LSTM) soft sensor for predicting effluent BOD, enhancing the prediction accuracy and efficiency. Firstly, the selection of appropriate auxiliary variables for soft-sensor modeling is determined through the calculation of k-nearest-neighbor mutual information (KNN-MI) values between the global process variables and effluent BOD. Subsequently, considering the existence of strong interactions among different reaction tanks, a Bi-LSTM neural network prediction model is constructed with historical data. Then, a multivariate probability density-based auto-reconstruction (MPDAR) strategy is developed for adaptive updating of the prediction model, thereby enhancing its robustness. Finally, the effectiveness of the proposed soft sensor is demonstrated through experiments using the dataset from Benchmark Simulation Model No.1 (BSM1). The experimental results indicate that the proposed soft sensor not only outperforms some traditional models in terms of prediction performance but also excels in avoiding ineffective model reconstructions in scenarios involving complex dynamic wastewater treatment conditions.

## 1. Introduction

With the rapid increase in population and the development of urbanization, more and more urban wastewater needs to be treated urgently [1,2]. The activated sludge mechanism, recognized as a convenient and efficient treatment technology, has seen widespread adoption in urban wastewater treatment plants in recent years [3]. Effluent biological oxygen demand (BOD) serves as a vital parameter for assessing discharge standards and indirectly reflects the amount of biodegradable organic matter in wastewater [4,5]. Therefore, timely and accurate prediction of effluent BOD is crucial as it can provide valuable insights for the safe operation and scientific management of wastewater treatment plants. At present, the commonly used detection methods encompass offline sampling and testing, online instrument monitoring, and the utilization of soft sensor technology [6]. Among these, soft sensor technology has garnered significant attention in recent years due to its convenient operation, low economic cost, and precise detection outcomes [7,8,9]. Notably, the development of storage and computational capabilities of hardware devices has propelled the use of data-driven soft sensors as a prominent detection method. Meng et al. proposed an adaptive task-oriented radial basis function (ATO-RBF) network to predict the effluent BOD through pruning the RBF nodes without sacrificing the learning accuracy [10]. To improve the prediction efficiency of effluent BOD, Pattnaik et al. proposed a cloud and edge layer collaborative soft sensor system architecture [11]. However, there are numerous variables in wastewater treatment processes, and the operational conditions constantly change due to internal and external factors; thus, how to improve the robustness of models while reducing time consumption of training processes is a worthwhile research topic.

For one thing, variable selection is a crucial step in the implementation of soft sensor technology, aimed at diminishing the dimensionality of sample data and simplifying model complexity [12]. The conventional methods for variable selection typically depend on mechanistic analysis and manual expertise to identify relevant process variables associated with BOD. However, the lack of a solid theoretical foundation often leads to imprecise selection. Then, principal component analysis (PCA) offers a means of dimensionality reduction through statistical examination of raw data, significantly easing the computational load in modeling [13]. However, PCA falls under unsupervised learning, resulting in reduced interpretability of the model due to the dimensionality-reduced variables that are not the original process variables. To address this problem, some researchers explored selecting the auxiliary variables depending on the Pearson correlation coefficient (PCC) [14] and mutual information (MI) [15]. Nonetheless, these approaches fail to consider spatial characteristics among variables in wastewater treatment processes and the constantly increasing computational burden.

For another, the utilization of a dependable prediction model is vital for enhancing the precision of soft sensor detection outcomes [16]. Presently, advanced deep learning algorithms like convolutional neural networks (CNNs) [17], graph neural networks (GNNs) [18], and recurrent neural networks (RNNs) [19] have gained substantial traction in wastewater treatment processes of variable prediction owing to their exceptional capacity for fitting complex data. Regrettably, the lengthy training duration of deep neural networks often relegates such soft sensors to offline modeling but online application [20]. At the same time, instances of abrupt alterations in external or internal conditions can severely compromise the robustness of the model, causing a significant decline in predictive accuracy. To solve this problem, scholars have directed their focus towards model correction and reconstruction [21,22,23]. For instance, Liu et al. proposed a method of structural pruning, involving iterative adjustments to the model architecture to accommodate dynamic condition changes [24]. Nevertheless, corrective measures tend to fall short when confronted with extreme scenarios. Therefore, Li et al. proposed the use of a moving window (MW) to continually update training data and reconstruct the prediction model [25]. However, the iterative reconstruction process demands extensive time for parameter fine-tuning and optimization, thereby impeding the timeliness of prediction results.

In response to the above issues, this paper proposed a multivariable probability density-based auto-reconstruction Bi-LSTM (MPDAR-Bi-LSTM) soft sensor for predicting effluent BOD in wastewater treatment plants. The main contributions and novelties of this study are as follows:

(1) To select auxiliary variables closely related to effluent BOD from global process variables for subsequent modeling, the k-nearest-neighbor (KNN) method is used instead of the original probability density computation method. This enhances the computational efficiency of mutual information (MI) values as well as incorporates an exploration of the spatial characteristic among process variables.

(2) Considering that each process variable in WWTP is affected by its own temporal changes, a prediction model is constructed leveraging the Bi-LSTM neural network, which consists of forward and backward LSTM layers to extract the latent information during data transmission.

(3) To enhance the robustness of the prediction model while ensuring its prediction efficiency, a multivariable probability density-based auto-reconstruction (MPDAR) strategy is proposed. It determines the necessity of model reconstruction by comparing the change rate of multivariate mathematical expectations, striking a balance between the stability and timeliness of the prediction model.

The rest of this paper is organized in accordance with the following scheme. In Section 2, we introduce the related works of KNN-MI for variable selection, Bi-LSTM for modeling prediction, and MPDAR strategy for model updating, which are the foundations of the proposed MPDAR-Bi-LSTM soft sensor. In Section 3, the proposed soft sensor is experimentally validated and analyzed on BSM1. Finally, the conclusions are given in Section 4, and some potential future research directions are provided.

## 2. Methodology

The main content of Section 2 includes the following four points. Section 2.1 provides a KNN-MI method to select auxiliary variables closely related to effluent BOD from global process variables for subsequent modeling, which enhances the computational efficiency of MI values as well as incorporates an exploration of the spatial characteristics among process variables. Section 2.2 introduces the theoretical knowledge of the Bi-LSTM neural network, which serves as the foundation for subsequent prediction models. Section 2.3 provides an MPDAR strategy that determines the necessity of prediction model reconstruction, which is the key to enhancing the robustness of the prediction model while ensuring its prediction efficiency. On the basis of all the theoretical knowledge and innovative methods mentioned above, the MPDAR-Bi-LSTM soft sensor for predicting effluent BOD in wastewater treatment plants is proposed. And Section 2.4 provides the specific steps of the proposed MPDAR-Bi-LSTM soft sensor depending on the previous statements.

### 2.1. KNN-MI for Variable Selection

Mutual information (MI) is a measure of the degree of interdependence between two variables [26]. The larger the MI value, the stronger both the linear and nonlinear correlations between two variables. Currently, it has been applied in various fields such as data science, information security, and process industry. According to its definition, the specific calculation formula is as follows:

(1) $$I\left(X,Y\right)=\;\int \int \;\rho \left(x,y\right)\mathrm{lg}\frac{\rho (x,y)}{\rho (x)\rho (y)}dxdy|x\in X,y\in Y$$

where ρ(x,y) represents the joint probability density value between two variables *x* and *y*. ρ(*x*) and ρ(*y*) are the marginal probability density values of variables *x* and *y*, respectively. I(X,Y) is the MI value between vectors *X* and *Y*.

In order to calculate the MI values more conveniently, this paper uses KNN to directly approximate their probability density values [27]. The main idea is to first identify k-nearest-neighbor samples, then find the number of other samples whose distances from the k-nearest-neighbor samples are less than the maximum distance between the current sample and the k-nearest-neighbor samples, and finally obtain the MI values through counting the numbers. The specific calculation formula is as follows:

(2) $$I\left(X,Y\right)=\Psi \left(x\right)-1/k-\langle \Psi \left(n_{x}\right)+\Psi \left(n_{y}\right)\rangle +\Psi \left(S\right)|x\in X,y\in Y$$

where Ψ(·) represents the digamma function. *k* is the number of nearest-neighbor samples, usually between 2 and 6. $\varepsilon_{i}/2$ is defined as the distance from the *i*-th sample $x_{i}$ or $y_{i}$ to the *k*-th-nearest-neighbor sample, so $n_{x}$ is the number of samples with a distance less than $\varepsilon_{i}/2$ from $x_{i}$; $n_{y}$ is the number of samples with a distance less than $\varepsilon_{i}/2$ from $y_{i}$. 〈·〉 is the average value of the digamma function for all variables, $\langle \cdot \rangle =\sum_{i=1}^{N}E\left(\cdot \right)/S$; *S* represents the number of samples.

KNN-MI can more conveniently identify the correlation between the target variable and other process variables. When given a suitable threshold λ, we can directly select the most appropriate variables for subsequent modeling.

### 2.2. Bi-LSTM for Modeling Prediction

**Figure 1:**
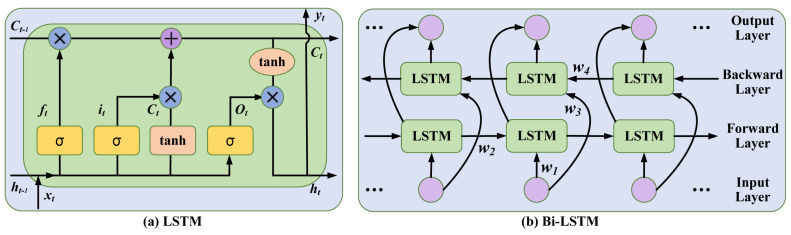
The structural diagrams of LSTM and Bi-LSTM.

Deep learning algorithms are an emerging method for solving the problem of variable prediction in modern industries. Notably, recurrent neural networks like LSTM stand out among other neural networks due to their distinctive “memory function” [28]. LSTM is usually composed of a forget gate, an input gate, and an output gate. The structural diagram is shown in Figure 1a. These components serve distinct purposes: the forget gate manages the retention or deletion of existing information, the input gate deals with receiving and storing new data, while the output gate regulates the output contributions of individual units.

In the forget gate, the process of information transmission is as follows:

(3) $$f_{t}=\sigma \left(\omega_{f_{h}}\left[h_{t-1}\right],\omega_{f_{x}}\left[x_{t}\right],b_{f}\right)$$

where $f_{t}$ represents the output of this gate, between 0 and 1. 0 indicates completely removing the value, and 1 implies reserving the whole value. σ is a sigmoid activation function. $\omega_{f_{h}}$ and $\omega_{f_{x}}$ are the weight matrices; $b_{f}$ is the bias vector of the forget gate. $h_{t-1}$ and $x_{t}$ are the output of the last moment and the input of the current time, respectively.

In the input gate, this gate consists of two layers: (1) a sigmoid layer, and (2) a “tanh” layer. The process of information transmission is as follows:

(4) $$\begin{array}{cc} & i_{t}=\sigma \left(\omega_{i_{h}}\left[h_{t-1}\right],\omega_{i_{x}}\left[x_{t}\right],b_{i}\right)\\ & {\tilde{C}}_{t}=\tanh\left(\omega_{c_{h}}\left[h_{t-1}\right],\omega_{c_{x}}\left[x_{t}\right],b_{c}\right) \end{array}$$

where $i_{t}$ is between 0 and 1, which represents the transmission proportion of ${\tilde{C}}_{t}$. ${\tilde{C}}_{t}$ is a newly added candidate status during the current moment. tanh is a commonly used activation function. $\omega_{i_{h}}$ and $\omega_{i_{x}}$ the weight matrices. $b_{i}$ and $b_{c}$ are the bias vectors of the input gate.

Afterwards, the current state $C_{t}$ is updated through the forget gate and input gate, the mathematical equation is as follows:

(5) $$\begin{array}{c} C_{t}=f_{t}\times C_{t-1}+i_{t}\times {\tilde{C}}_{t} \end{array}$$

where $f_{t}$ represents the output of the forget gate, $i_{t}$ represents the transmission proportion of ${\tilde{C}}_{t}$, and $C_{t-1}$ represents the previous state.

In the output gate, the process of information transmission is as follows:

(6) $$\begin{array}{cc} & O_{t}=\sigma \left(\omega_{o_{h}}\left[h_{t-1}\right],\omega_{o_{x}}\left[x_{t}\right],b_{o}\right)\\ & h_{t}=O_{t}\times \tanh\left(C_{t}\right) \end{array}$$

where $O_{t}$ is the output of the output gate, between 0 and 1. $h_{t}$ represents the final output value. $\omega_{o_{h}}$ and $\omega_{o_{x}}$ are the weight matrices, and $b_{f}$ is the bias vector of the forget gate.

Bi-LSTM is an extension of LSTM, which consists of two layers of LSTM networks: one for forward propagation and the other for backward propagation [29]. Two layers can improve learning long-term dependencies. The structural diagram of Bi-LSTM is shown in Figure 1b.

In the forward propagation layer, the process of information transmission is as follows:

(7) $$\begin{array}{c} L_{t}^{f}=\sigma \left(\omega_{1}\left[x_{t}\right],\omega_{2}\left[L_{t-1}^{f}\right],b_{L^{f}}\right) \end{array}$$

In the backward propagation layer, the process of information transmission is as follows:

(8) $$\begin{array}{c} L_{t}^{b}=\sigma \left(\omega_{3}\left[x_{t}\right],\omega_{4}\left[L_{t-1}^{b}\right],b_{L^{b}}\right) \end{array}$$

where $L_{t}^{f}$ and $L_{t}^{b}$ are the outputs of the forward propagation layer and backward propagation layer, respectively. $\omega_{1}$, $\omega_{2}$, $\omega_{3}$, and $\omega_{4}$ are the weight matrices. $b_{L^{f}}$ and $b_{L^{b}}$ are bias vectors.

In this paper, we constructed a Bi-LSTM-based soft sensor for variable prediction. Benefiting from the bidirectional information transmission process, Bi-LSTM is capable of revealing latent information and simulating the wastewater treatment process, consequently enhancing the prediction performance of the model.

### 2.3. MPDAR Strategy for Model Updating

Probability density is a statistical information measurement that serves as a valuable tool for characterizing the distribution properties of sample data. For the convenience of solving, we choose kernel density estimation (KDE) to calculate the probability density value of each process variable [30]. The KDE formula is as follows:

(9) $$\begin{array}{c} p\left(x\right)=\frac{1}{nh}\sum_{i=1}^{n}\phi (-\frac{x_{i}-x}{h}) \end{array}$$

where p(·) represents the probability density value. *n* is the number of output data. *h* is a hyperparameter named window width. ϕ(·) is a Gaussian kernel function, defined as follows:

(10) $$\begin{array}{c} \phi \left(x\right)=\frac{1}{\sqrt{2\pi }}e^{-\frac{{∥x∥}^{2}}{2}} \end{array}$$

Then, we calculate the mathematical expectation of vector *X*, denoted as E(X). The formula for calculating E(X) is as follows:

(11) $$\begin{array}{c} E\left(X\right)=\int_{-\infty }^{\infty }x\cdot p\left(x\right)dx \end{array}$$

Finally, the change rate of E(X) is calculated as follows:

(12) $$\begin{array}{c} J=\left|\frac{E\left(X_{t}\right)-E\left(X_{t-1}\right)}{E(X_{t-1})}\right|\times 100\% \end{array}$$

We extend Equation (11) to multivariate systems, and the calculation formula of *J* is as follows:

(13) $$\begin{array}{c} J=\left|\frac{\sum_{i=1}^{m}W_{i}\cdot (\left(E_{i}\left(X_{t}\right)-E_{i}\left(X_{t-1}\right)\right)}{\sum_{i=1}^{m}\left(W_{i}E_{i}\left(X_{t-1}\right)\right)}\right|\times 100\% \end{array}$$

where $W_{i}$ represents the weight value, which is determined by relying on the previous KNN-MI values. The specific calculation formula for $W_{i}$ is as follows:

(14) $$\begin{array}{c} W_{i}=\frac{I(X_{i},Y)}{\sum_{i=1}^{m}I\left(X_{i},Y\right)} \end{array}$$

where $I(X_{i},Y)$ is the KNN-MI value between $X_{i}$ and *Y*. *m* is the number of auxiliary variables.

Comparing with the provided threshold, if *J* surpasses the designated threshold, it signifies a noteworthy alteration in the distribution attributes of the training data, necessitating a reconstruction of the model. Conversely, if *J* falls below the threshold, there is no imperative for model reconstruction. Therefore, selective reconstruction of the model can be applied instead of blind reconstruction.

### 2.4. The Proposed MPDAR-Bi-LSTM Soft Sensor

**Figure 2:**
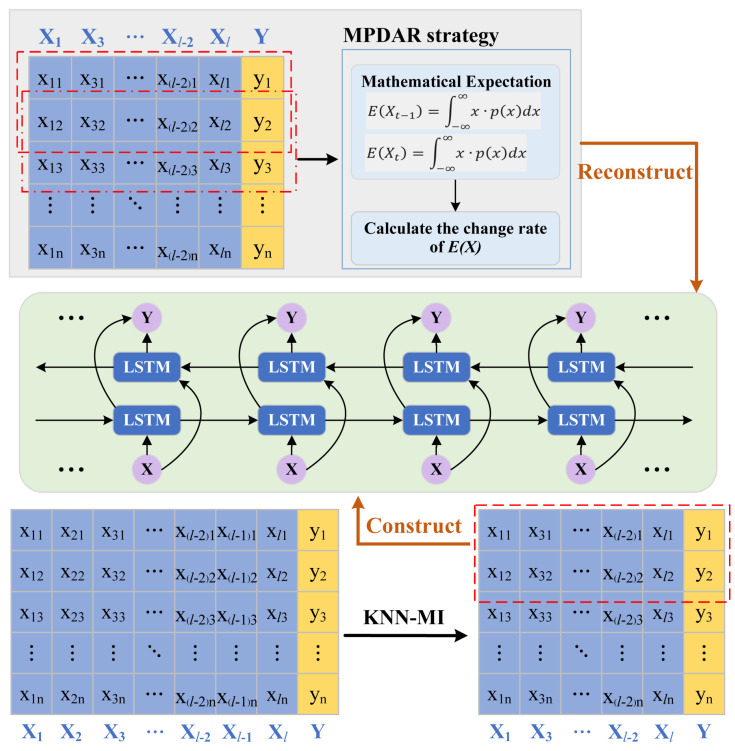
The algorithm flowchart of the proposed MPDAR-Bi-LSTM soft sensor.

This subsection provides the specific steps of the proposed MPDAR-Bi-LSTM soft sensor depending on the previous statements. Figure 2 shows the algorithm flowchart of the proposed soft sensor. The outlined steps are as follows.

**Step 1**: Collect sample data from the target WWTP and perform preprocessing of the collected data.

**Step 2**: Calculate the KNN-MI values between each process variable and effluent BOD, and then select the auxiliary variables for subsequent modeling according to the given threshold λ.

**Step 3**: Construct a Bi-LSTM-based prediction model using the historical training data.

**Step 4**: Calculate the change rate of mathematical expectation of the training dataset before and after updates, and determine whether to reconstruct the prediction model.

**Step 5**: Use the final model to predict the effluent BOD value of the test data, and assess the accuracy through comparing with the real values.

## 3. Experiments

### 3.1. Background Description

**Figure 3:**
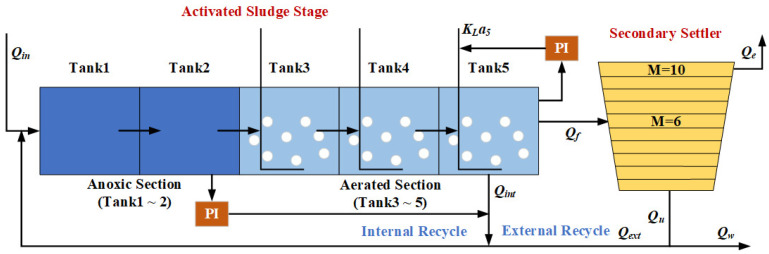
The basic structure of the BSM1 plant.

BSM1 is a universal wastewater treatment simulation platform designed by the International Water Association (IWA), enabling researchers to conduct experiments on a unified platform [31]. BSM1 simulates the modern urban wastewater treatment process, mainly the removal of nitrogen-containing organic matter. Figure 3 shows the basic structure of BSM1, which consists of a five-compartment activated sludge reactor (two anoxic tanks followed by three aerobic tanks) and a secondary settling tank. Different operation conditions are simulated by changing the influent data of the platform, such as during sunny days, rainy days, and rainstorm days.

In this case, to verify the stability of the proposed soft sensor prediction performance, we collected experimental data under different weather conditions into a complex dataset containing multiple operation conditions. This dataset comprised data gathered from five sunny weekdays, a sunny weekend, two rainy days, and three rainstorm days. The process variables were collected at different stages including water inlet, reaction tanks (tank 1 to tank 5), secondary settler, recycle, and water outflow. An introduction to the specific variables is provided in Table 1. The sampling period for each variable was 15 min, which clearly reflects the changes in pollutants and treatment efficiency. We used the data from the first three sunny days (288 samples) as initial training data, and the remaining nine days of data (864 samples) as test data to assess the efficacy of the models.

**Figure 4:**
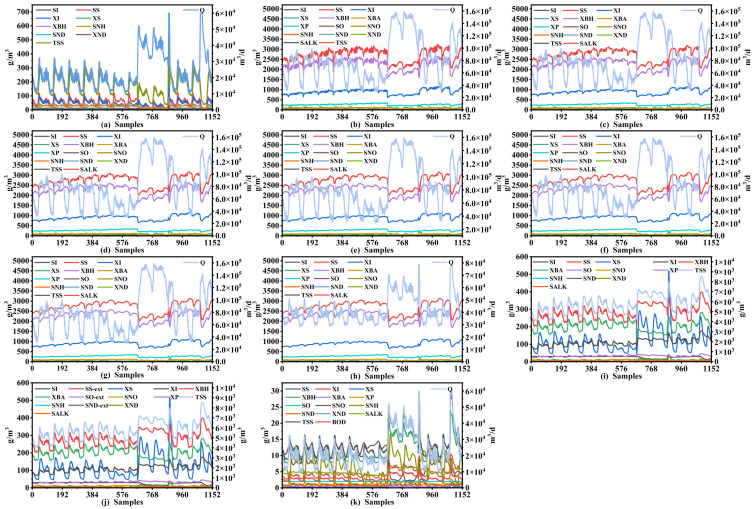
The curves of process variables in different stages of the BSM1 plant. (**a**) Original inflow of the plant; (**b**) input of reaction tank 1; (**c**) output of reaction tank 1; (**d**) output of reaction tank 2; (**e**) output of reaction tank 3; (**f**) output of reaction tank 4; (**g**) output of reaction tank 5; (**h**) input of the secondary settler; (**i**) underflow of the secondary settler; (**j**) external recycle; (**k**) outflow of the plant.

Figure 4 illustrates the curves of the process variables of different treatment stages in the WWTP, including input port, reaction tank 1, reaction tank 2, reaction tank 3, reaction tank 4, reaction tank 5, and secondary settler. We can see that corresponding fluctuations occur in the process variables in the subsequent treatment stages simultaneously due to variations in the influent data. For example, two obvious mutation points in the influent data are located at the 673rd and 870th sample points, and corresponding changes occur in the subsequent treatment stages at the same sample points. Furthermore, we find that preprocessing of the collected data can effectively eradicate the problem of time delay. In other words, these process variables sampled at different locations can reflect the wastewater treatment information at the same moment.

### 3.2. Variable Selection Results

**Figure 5:**
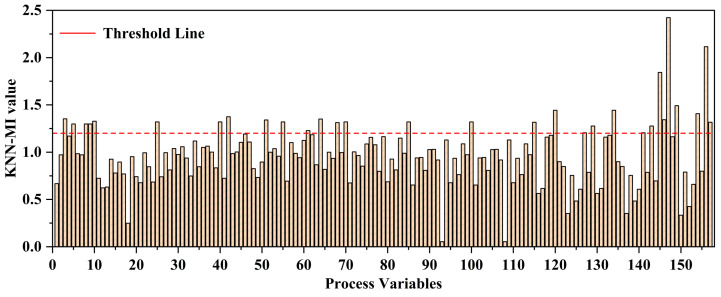
The KNN-MI values between each process variable and effluent BOD.

In this case, we collected a total of 157 process variables, which cover various stages of the global wastewater treatment process. Utilizing all these variables for modeling would lead to a significant increase in model complexity and could adversely impact the prediction accuracy due to the accumulation of redundant information. Therefore, following the application of KNN-MI for variable selection, we selected 22 auxiliary variables from the 157 process variables. The selected auxiliary variables have strong correlations with effluent BOD, which can meet the accuracy requirements for predicting effluent BOD. At the same time, it reduces the complexity of the data dimensions and soft sensing models, eliminates information redundancy caused by weakly correlated variables, and is of great significance for improving the predictive performance of the model. Figure 5 shows the KNN-MI values between each process variable and effluent BOD, with a threshold line of λ=1.2. When the KNN-MI value exceeds 1.2, it means that the variable has a strong correlation with the effluent BOD. On the contrary, when the KNN-MI value is below this threshold line, the variable is removed.

### 3.3. Evaluation Indicators

To verify the predictive performance of the proposed soft sensor, root mean square error (RMSE), mean absolute error (MAE), and Pearson correlation coefficient (PCC) are selected as evaluation metrics [32]. The specific formulas are as follows:

(15) $$\begin{array}{c} RMSE=\frac{1}{N}\sqrt{\sum_{i=1}^{N}{\left({\hat{y}}_{i}-y_{i}\right)}^{2}} \end{array}$$

(16) $$\begin{array}{c} MAE=\frac{1}{N}\sum_{i=1}^{N}\left|{\hat{y}}_{i}-y_{i}\right| \end{array}$$

(17) $$\begin{array}{c} PCC\left(Y,\hat{Y}\right)=\frac{Cov(Y,\hat{Y})}{\sqrt{Var\left(Y\right)\cdot Var\left(\hat{Y}\right)}} \end{array}$$

where *N* represents the number of test data. $y_{i}$ and ${\hat{y}}_{i}$ are the real value and predictive value of the *i*-th sample. *Y* and $\hat{Y}$ are the real and predictive datasets. Cov(·) is the covariance and Var(·) is the variance.

### 3.4. Prediction Results and Analysis

**Figure 6:**
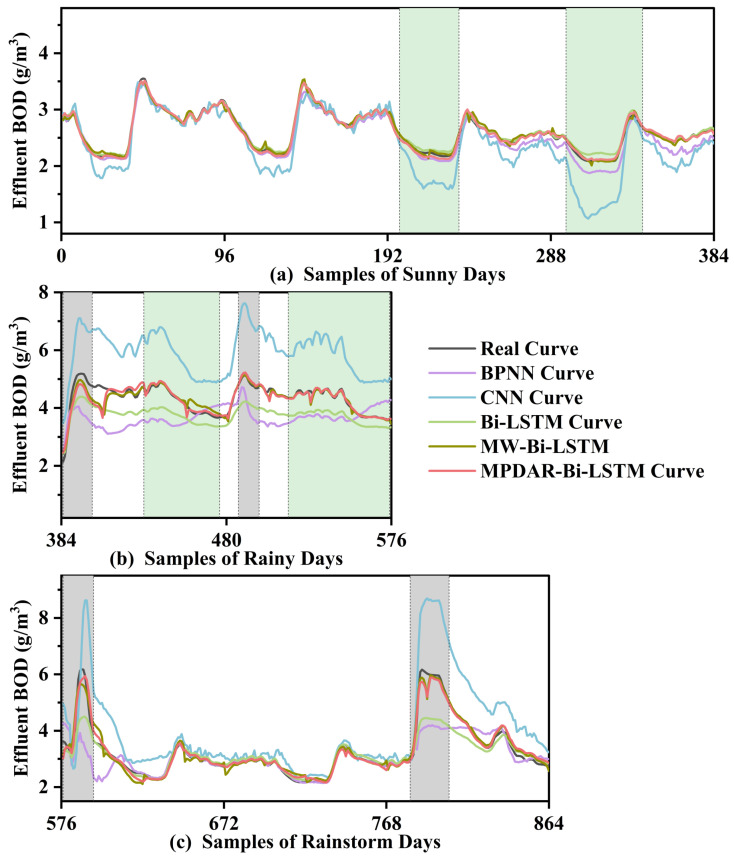
Prediction results of effluent BOD in the WWTP under (**a**) sunny days, (**b**) rainy days, and (**c**) rainstorm days. (For interpretation of the colors in this figure, the reader is referred to the web version of this article.)

The global performance evaluation indicators of the BPNN, CNN, Bi-LSTM, MW-Bi-LSTM, and the proposed MPDAR-Bi-LSTM models are shown in Table 2. Firstly, for all the five models, it can be seen that the prediction accuracy of the three models based on Bi-LSTM is far superior to BPNN and CNN, while the time consumption is longer than CNN and shorter than BPNN. Secondly, for Bi-LSTM, MW-Bi-LSTM, and the proposed MPDAR-Bi-LSTM models, the traditional Bi-LSTM model exhibits inferior predictive performance compared to the other two models. This disparity is primarily ascribed to the inherent characteristics of Bi-LSTM as a prevalent deep neural network, typically employed for offline training. In the face of frequent variations in conditions prompted by an intricate external environment, offline models often demonstrate limited adaptability, particularly in instances of significant fluctuations such as inclement weather conditions. In contrast, the MW-Bi-LSTM and the proposed MPDAR-Bi-LSTM models demonstrate notably enhanced predictive capabilities compared to the Bi-LSTM model. This can be attributed to their capacity for sustaining robust model performance through continual model reconstruction. Nonetheless, it is essential to acknowledge that the reconstruction process leads to increased time consumption. As illustrated in Table 2, the time consumption of the two models is 1068.31 s and 638.47 s, respectively, significantly exceeding the 34.65 s recorded for the Bi-LSTM model. However, for the two prediction methods with the ability to update the model, the MPDAR-Bi-LSTM model has a selective update mechanism due to the MPDAR strategy, unlike the MW-Bi-LSTM model. For example, as shown in Figure 6a, the 1-96 test samples on sunny days have similar distribution characteristics with the initial training data; the change rate of E(X) is *J*, calculated using the MPDAR strategy, and *J* does not surpass the designated threshold, so there is no need to update the MPDAR-Bi-LSTM prediction model. But the MW-Bi-LSTM model lacks an analysis of the data distribution characteristics and continuously updates the prediction model. So, the proposed MPDAR-Bi-LSTM model is clearly more efficient. Therefore, in summary, the proposed MPDAR-Bi-LSTM model has higher model accuracy and stronger robustness compared to the traditional Bi-LSTM model, BPNN model, and CNN model, and has better overall performance than the MW-Bi-LSTM model, while consuming less time.

In order to compare the prediction results of the above-mentioned five different models more intuitively, Figure 6 shows the fitting result between the predictive curve and the real curve of effluent BOD in WWTP under different weather conditions; Table 3 shows the performance evaluation indicators of the five models under different weather conditions. As shown in Figure 6a, the first two days are sunny weekdays, and the process data are close to the initial training data. The prediction performance of BPNN, Bi-LSTM, MW-Bi-LSTM, and the proposed MPDAR-Bi-LSTM models is good, but the prediction error of the CNN model is a little large. The last two days are sunny weekend days, and there are slight changes in the working conditions compared to sunny weekdays. The prediction performance of the BPNN and CNN models deteriorates. Due to the inability to update the prediction model, the prediction performance of the Bi-LSTM model is slightly worse than the MW-Bi-LSTM and the MPDAR-Bi-LSTM models, as shown in the green shaded area. According to the performance indices in Table 3, it can be seen that the MPDAR-Bi-LSTM model has better overall performance compared to the MW-Bi-LSTM model. As shown in Figure 6b, there were significant changes in the working conditions on rainy days compared to sunny weekdays. The BPNN, CNN, and Bi-LSTM models had a large prediction error due to their inability to update the model, while the MW-Bi-LSTM model and the MPDAR-Bi-LSTM model were able to update the prediction model in a timely manner, achieving better prediction results. However, upon further analysis, the MW-Bi-LSTM model could make corresponding adjustments more quickly after switching operating conditions compared to the MPDAR-Bi-LSTM model, as shown in the gray shaded area of Figure 6b. Although the adjustment time of the MPDAR-Bi-LSTM model is slightly longer, the final prediction result is better than the MW-Bi-LSTM model, as shown in the green shaded area in Figure 6b. Finally, as shown in Figure 6c, the working conditions on rainstorm days changed more than those on sunny weekdays. The BPNN, CNN, and Bi-LSTM models cannot update the model, leading to significant prediction errors. The MW-Bi-LSTM model and the MPDAR-Bi-LSTM model can update the prediction model in time to achieve better prediction results. Different from the previous two situations, the change in working conditions on rainstorm days is more extreme. In the early stage, the fast tracking performance of the MPDAR-Bi-LSTM model is slightly weaker than that of MW-Bi-LSTM, as shown in the gray shaded area of Figure 6c. But as time passes, the adjusted MPDAR-Bi-LSTM model gradually achieved more accurate prediction results. In addition, under the gentle working conditions between the two extreme rainstorm events, the tracking performance of the MPDAR-Bi-LSTM model is better than that of MW-Bi-LSTM. Therefore, the proposed MPDAR-Bi-LSTM model has the best comprehensive performance under normal working conditions, unstable working conditions, and extreme events.

The experimental results show that the proposed MPDAR-Bi-LSTM soft sensor exhibits the capability to optimize the prediction precision and efficiency in the face of complex wastewater treatment processes. This achievement is primarily attributed to the MPDAR strategy. This strategy calculates the statistical mean values of various procedural variables and integrates them according to the KNN-MI values, thereby offering a more thorough portrayal of operational conditions of wastewater treatment plants. Then, by leveraging the variation rate of the mathematical expectation, it determines whether the operational conditions have changed, and selectively reconstructs the model. However, it is not difficult to find that even though the proposed MPDAR strategy largely avoids ineffective updates, it still requires a significant amount of time cost contrasted with offline models. Moreover, when faced with extreme changes, new models perform slightly poorly in predicting extreme values during the initial prediction phases. Finally, the "memory function" of Bi-LSTM neural networks may sometimes transmit negative information, thereby influencing the accuracy of prediction performance.

## 4. Conclusions

In order to improve the prediction accuracy of effluent BOD in the wastewater treatment process and ensure the timeliness of prediction results, this paper proposed an MPDAR-Bi-LSTM soft sensor. The proposed soft sensor firstly uses KNN-MI to select appropriate process variables for modeling, thereby reducing data dimensionality. Then, a Bi-LSTM neural network with “memory function” is used to construct the prediction model. Afterwards, to enhance model robustness and prevent ineffective model reconstruction, a novel MPDAR strategy is developed to update the model. Finally, experiments are conducted based on the dataset, with complex operational conditions, collected from BSM1. The experimental results show that compared with BPNN and CNN methods, the overall predictive performance of methods based on Bi-LSTM is better because each process variable in the wastewater treatment processes is affected by its own temporal changes and the prediction models based on Bi-LSTM can better extract the latent information during data transmission. As for the three methods based on Bi-LSTM, compared to the traditional Bi-LSTM method, the proposed MPDAR-Bi-LSTM model significantly improves the prediction accuracy. And compared with the MW-Bi-LSTM method, although the adjustment time of the proposed model is slightly longer, the total time consumption is reduced by 40.24% and the RMSE and MAE of the final prediction result is improved by 4.92% and 9.51%, respectively. To sum up, the proposed MPDAR-Bi-LSTM model has higher model accuracy and stronger robustness compared to the traditional BPNN, CNN, and Bi-LSTM models, and has better overall performance than the MW-Bi-LSTM model, while consuming less time. But then again, it cannot be denied that there is still a lot of room for improvement in the proposed method, and future research directions may include exploring large-scale models and diverse adaptive algorithms.

## Figures and Tables

**Table 1:** The notation and definition of process variables and system components.

Notation	Definition	Notation	Definition
SI	Soluble inert organic matter	XI	Particulate inert organic matter
SS	Readily biodegradable substrate	XS	Slowly biodegradable substrate
SO	Oxygen	XBH	Active heterotrophic biomass
SNO	Nitrate and nitrite nitrogen	XBA	Active autotrophic biomass
SNH	$NH_{4}^{+}+NH_{3}$ nitrogen	XP	Particulate products arising from biomass decay
SND	Soluble biodegradable organic nitrogen	XND	Particulate biodegradable organic nitrogen
SALK	Alkalinity	TSS	Total readily biodegradable substrate
Q	Flow rate	BOD	Biochemical oxygen demand
in	Water inlet	bef	Summary input before tank 1
r1	Reaction tank 1	int	Internal recycle
r2	Reaction tank 2	feed	Input of the secondary settler
r3	Reaction tank 3	ext	External recycle
r4	Reaction tank 4	under	Underflow
r5	Reaction tank 5	out	Water outflow

**Table 2:** The global performance evaluation indicators of the five different models.

Model	RMSE (g/m^3^)	MAE (g/m^3^)	PCC	Time (s)
**BPNN**	0.5769	0.3260	0.1649	12.63
**CNN**	0.9973	0.7137	0.6704	35.39
**Bi-LSTM**	0.4023	0.2269	0.5556	34.65
**MW-Bi-LSTM**	0.1464	0.0831	0.9736	1068.31
**MPDAR-Bi-LSTM**	0.1392	0.0752	0.9762	638.47

**Table 3:** The performance evaluation indicators of the five different models under different conditions.

Method	Sunny Days			Rainy Days			Rainstorm Days		
	RMSE ^1^	MAE ^2^	PCC ^3^	RMSE	MAE	PCC	RMSE	MAE	PCC
**BPNN**	0.1049	0.0815	0.9185	0.9157	0.8215	−6.5879	0.6517	0.3216	−0.2092
**CNN**	0.3456	0.2547	0.6066	1.6136	1.5819	−3.3784	1.0434	0.7468	0.5724
**Bi-LSTM**	0.0384	0.0323	0.9995	0.7602	0.5928	0.9652	0.4756	0.2423	0.9673
**MW-Bi-LSTM**	0.0356	0.0300	0.9996	0.2458	0.1198	0.9973	0.1875	0.1295	0.9957
**MPD-Bi-LSTM**	0.0307	0.0284	0.9997	0.2623	0.1212	0.9969	0.1608	0.1068	0.9968

^1^ The units of RMSE are g/m^3^. ^2^ The units of MAE are g/m^3^. ^3^ PCC is a dimensionless coefficient.

## Data Availability

Data are contained within the article.
